# Impact of Microbiome–Brain Communication on Neuroinflammation and Neurodegeneration

**DOI:** 10.3390/ijms241914925

**Published:** 2023-10-05

**Authors:** Iris Stolzer, Eveline Scherer, Patrick Süß, Veit Rothhammer, Beate Winner, Markus F. Neurath, Claudia Günther

**Affiliations:** 1 Department of Medicine 1, Universitätsklinikum Erlangen, Friedrich-Alexander-Universität Erlangen-Nürnberg (FAU), 91054 Erlangen, Germany; 2 Department of Molecular Neurology, Universitätsklinikum Erlangen, Friedrich-Alexander-Universität Erlangen-Nürnberg (FAU), 91054 Erlangen, Germany; 3 Department of Neurology, Universitätsklinikum Erlangen, Friedrich-Alexander-Universität Erlangen-Nürnberg (FAU), 91054 Erlangen, Germany; 4 Department of Stem Cell Biology, Universitätsklinikum Erlangen, Friedrich-Alexander-Universität Erlangen-Nürnberg (FAU), 91054 Erlangen, Germany; 5 Center of Rare Diseases Erlangen (ZSEER), Universitätsklinikum Erlangen, Friedrich-Alexander-Universität Erlangen-Nürnberg (FAU), 91054 Erlangen, Germany; 6 Deutsches Zentrum Immuntherapie (DZI), Universitätsklinikum Erlangen, 91054 Erlangen, Germany

**Keywords:** neurodegenerative diseases, microbial dysbiosis, gut–brain-axis

## Abstract

The gut microbiome plays a pivotal role in maintaining human health, with numerous studies demonstrating that alterations in microbial compositions can significantly affect the development and progression of various immune-mediated diseases affecting both the digestive tract and the central nervous system (CNS). This complex interplay between the microbiota, the gut, and the CNS is referred to as the gut–brain axis. The role of the gut microbiota in the pathogenesis of neurodegenerative diseases has gained increasing attention in recent years, and evidence suggests that gut dysbiosis may contribute to disease development and progression. Clinical studies have shown alterations in the composition of the gut microbiota in multiple sclerosis patients, with a decrease in beneficial bacteria and an increase in pro-inflammatory bacteria. Furthermore, changes within the microbial community have been linked to the pathogenesis of Parkinson’s disease and Alzheimer’s disease. Microbiota–gut–brain communication can impact neurodegenerative diseases through various mechanisms, including the regulation of immune function, the production of microbial metabolites, as well as modulation of host-derived soluble factors. This review describes the current literature on the gut–brain axis and highlights novel communication systems that allow cross-talk between the gut microbiota and the host that might influence the pathogenesis of neuroinflammation and neurodegeneration.

## 1. Introduction

Neurologic and psychiatric disorders are a diverse group of conditions that affect the nervous system, including the central nervous system (CNS) and the enteric neural system (ENS). These disorders can result from various causes, including genetic, infectious, traumatic, and environmental factors. Alzheimer’s disease (AD), Parkinson’s disease (PD), and multiple sclerosis (MS) are among the most common ones. These disorders can cause a range of symptoms, including cognitive impairment, motor symptoms, and a plethora of other systems being affected [1,2,3]. These are caused both by neurodegeneration and neuroinflammation. Neurodegeneration refers to the gradual and progressive loss of structure or function of neurons. On the other hand, neuroinflammation is defined as an inflammatory response associated with autoimmunity, innate immune processes, or infections in the nervous system, leading to the production and release of cytokines, chemokines, and secondary messengers. Both neurodegeneration and neuroinflammation are tightly interconnected [1,2,3]. Extensive research has been conducted on the interaction between neurodegeneration and neuroinflammation, and the relationship between them is becoming increasingly clear. Recently, there has been a growing interest in the role of the gastrointestinal (GI) tract, including the gut microbiome and the mucosal immune system, in the development of neurodegenerative disorders and neuroinflammation. However, the role of both pathogenic and commensal microbes residing within the gut lumen in facilitating this cross-talk has only recently been appreciated. Studies have demonstrated the pivotal role of the gut microbiome in regulating the mucosal and systemic immune system, ultimately influencing inflammatory dynamics. Accordingly, disturbances in the gut microbiome have been implicated in contributing to the development of neurological disorders [4,5]. Consequently, extensive research has highlighted the gut–brain axis, a bidirectional communication network linking the GI tract and the CNS. This axis allows for the transfer of molecules, signals, and information between the brain and the gut, which can impact various physiological and psychological processes [6,7]. Communication along the gut–brain axis includes various neural, hormonal, metabolic, and immunological signals released by cells of the gut mucosa, including mucosal immune cells (lamina propria cells), intestinal epithelial cells that form the intestinal barrier, cells of the ENS, and other stromal cells of the gut microenvironment [8,9,10]. The vagus nerve—the main component of the parasympathetic nervous system—is also involved in the communication along the gut–brain axis, as its afferent fibers are able to respond to the microbiota indirectly via microbial metabolites and cytokines and transmit this information from the gut to the CNS to generate a response [11]. Finally, the gut–brain axis is complemented by the intestinal microbiota together with its metabolites and soluble products (the reason why this axis is often referred to as the microbiota–gut–brain axis) [8,12,13]. Further research is needed to fully elucidate the mechanisms underlying the so-called gut–brain axis and to develop effective therapeutic interventions.

**Figure 1:**
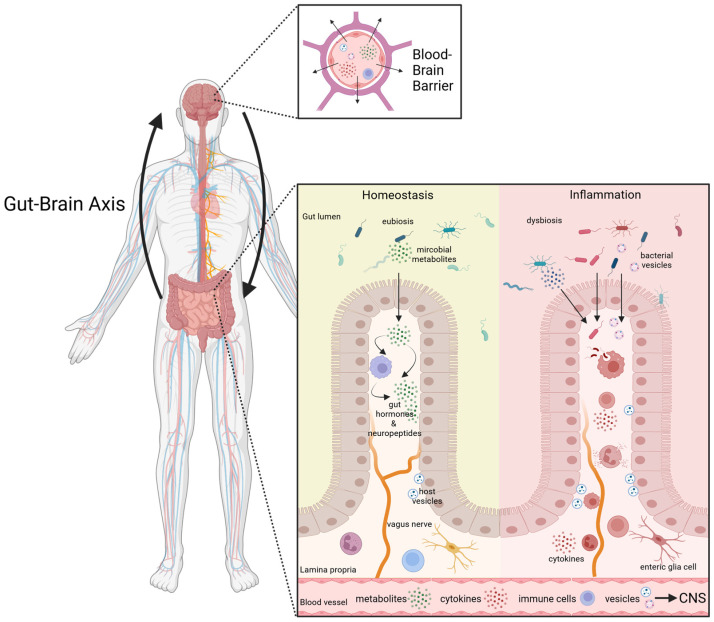
The Gut–Brain Axis. Emerging evidence from both clinical and experimental studies strongly indicates that the gut–brain axis plays a pivotal role in linking inflammatory conditions of the gut and the CNS. It is proposed that intestinal dysbiosis, along with the translocation of bacterial membrane products, diverse metabolites (e.g., SCFA, neuropeptides), as well as host-derived inflammatory factors in response to bacteria, exerts multifaceted influences on various aspects along this network. Factors can shuttle ether via the bloodstream or the vagus nerve and can cross the blood–brain barrier. Furthermore, a pro-inflammatory intestinal environment, often associated with increased intestinal permeability (“leaky gut”), may result in an altered communication along the gut–brain axis, potentially contributing to neuroinflammation in the ENS and CNS. Arrows indicate the bi-directional communication network linking the GI tract and the CNS. Graphic created with Biorender.com (accessed on 18 September 2023).

This review will summarize the current literature on the gut–brain axis (Figure 1), with a particular focus on microbial alterations and bacterial vesicles as novel communication systems that allow cross-talk between the gut/microbiota and the CNS in the context of AD, PD, as well as MS.

## 2. The Gut and the Brain—Neurological Disorders and Intestinal Inflammation

### 2.1. Multiple Sclerosis

Multiple sclerosis is a chronic autoimmune disease that affects the CNS and is characterized by inflammation, demyelination, and neurodegeneration. The pathogenesis of MS is multifactorial and involves genetic susceptibility, as well as environmental factors [14,15]. This neuroinflammatory disorder can be classified into different subtypes based on the clinical course of the disease, including relapsing-remitting MS (RRMS), secondary progressive MS (SPMS), and primary progressive MS (PPMS) [15,16]. The pathogenesis of MS is complex and involves immune-mediated damage to myelin, oligodendrocytes, and axons in the CNS. Activated T cells with an inflammatory phenotype, including CD4^+^ type 1 T helper (Th1) and Th17 (interleukin-17-producing T helper) cells, as well as CD8^+^ T cells, play a key role in driving the inflammatory response, while regulatory T cells (Tregs) can limit inflammation [17,18]. These important features of T helper cell-mediated autoimmunity are also represented in the experimental autoimmune encephalomyelitis (EAE), the most commonly used mouse model for MS. EAE is characterized by infiltration of T cells and monocytes into the CNS, leading to local inflammation. EAE is typically induced either by active immunization with myelin-derived proteins or peptides in an adjuvant or by passive transfer of activated myelin-specific CD4+ T cells, and it represents a suitable model to study potential treatments and the important aspects of the pathogenesis of MS [19]. Moreover, further cells involved in MS pathogenesis include B cells and glial cells, such as microglia and astrocytes, which contribute to inflammation, demyelination, and neurodegeneration. Disruption of the blood–brain barrier (BBB) is also a critical component of disease onset, allowing infiltration of peripheral immune cells into the CNS. So far, it is poorly understood how inflammation in the CNS is initiated; however, it is clear that the breakdown of the BBB is one of the critical components in disease onset [17,18]. While there is currently no cure for MS, several disease-modifying therapies are available that can slow disease progression and reduce the frequency and severity of relapses. Moreover, early diagnosis and treatment of MS are important to improve outcomes and prevent disability [16,20]. Of note, recent studies have highlighted a strong connection between gut inflammation and MS [7,17]. Several studies have described that MS patients display an increased prevalence of mucosal inflammation, including alterations in gut microbial composition and intestinal permeability [21,22]. Microbial dysbiosis may contribute to the activation of immune cells and inflammation in the CNS and is linked to the development of MS in humans and pre-clinical animal models [21,22,23,24]. The alterations of a Th17 immune response observed in MS patients were associated with changes within the microbial community and high disease activity [21,24]. But also, the production of IL-10, with its immune modulatory function, can be altered by the intestinal microbiota, and this relation is linked to CNS autoimmunity [23]. Additionally, various environmental risk factors for MS, such as diet, are known to modulate the intestinal microbiota and their metabolites and subsequently influence intestinal homeostasis [25,26]. Recent studies in microbial endocrinology have highlighted the role of neuroactive molecules, such as neurotransmitters, produced by gut microbes as an important aspect of the communication between the gut and the brain. These neurotransmitters, such as γ-aminobutyric acid (GABA), produced by specific bacterial species, can, directly and indirectly, influence brain cell physiology. GABA plays a vital role in balancing inhibitory and excitatory functions in the brain, suppressing cytokine release from pro-inflammatory immune cells, and regulating neuropeptide secretion in intestinal nerve fibers [27,28,29]. GABA treatment has shown effectiveness in reducing inflammation and improving conditions in animal models for MS [30]. Additionally, gut microbial-derived metabolites, such as SCFAs, are involved in neurophysiological, biochemical, and microbiological processes that may contribute to neurodegeneration and inflammation. Significantly noteworthy is the striking decline observed in species that are responsible for SCFA production, such as *Butyricimonas*, among MS patients. There is increasing evidence that microbiota-derived metabolites can serve as important regulators of the intestinal barrier, as well as the blood–brain barrier [31,32,33,34,35,36,37]. Overall, the communication between the gut and the CNS is strongly modulated by the enteric microbiota. Molecular mechanisms by which the gut microbiota influences MS pathogenesis and potential targets of this communication might represent promising novel avenues for therapeutic strategies for MS, but further research is needed to fully understand the role of the gut–brain axis [38,39,40].

### 2.2. Alzheimer’s Disease

In addition to MS, the intestinal microbiota has been implicated in the pathogenesis of Alzheimer’s disease. AD is a neurodegenerative disorder that primarily affects the elderly population and is characterized by progressive memory impairment, cognitive decline, and behavioral changes. It is the most common cause of dementia worldwide, with a rapidly increasing prevalence [41]. This multifactorial disease has a complex interplay between genetic and environmental factors contributing to its pathogenesis. The pathological hallmarks of AD include the accumulation of amyloid-beta (Aβ) plaques and neurofibrillary tangles composed of hyperphosphorylated tau protein, as well as neuroinflammation and synaptic dysfunction. The accumulation of Aβ peptides, resulting from the aberrant cleavage of the amyloid precursor protein (APP), is potentially toxic to neurons and crucial for AD pathogenesis [42,43,44]. Presently, clinical trials with antibodies targeting Aβ as disease-modifying therapies display promising results, whereas the majority of the existing therapies merely offer relief from AD symptoms. The ongoing endeavors in research are directed toward the creation of disease-modifying treatments that possess the capability to either halt or decelerate the advancement of AD [45,46,47]. Several studies have suggested a possible link between AD and intestinal inflammation. It has been shown that AD patients have an altered gut microbiota composition, with an increased abundance of pro-inflammatory bacteria and a decrease in anti-inflammatory bacteria [48,49]. This microbial dysbiosis may lead to increased intestinal permeability, allowing the translocation of bacterial products, such as lipopolysaccharides (LPS), into the bloodstream. LPS has been shown to activate microglia and induce neuroinflammation, which is an additional hallmark of AD pathology [50,51]. AD patients have been found to have increased levels of inflammatory cytokines, such as interleukin-6 (IL-6) and tumor necrosis factor-alpha (TNF), in both the brain and the blood, which may be linked to mucosal inflammation [52,53]. Further studies have also shown that targeting microbial dysbiosis with prebiotics or probiotics may have a beneficial effect on AD symptoms [54]. For example, it has been shown that supplementing AD patients with a probiotic mix containing *Lactobacillus* and *Bifidobacterium* resulted in improved cognitive functions and reduced levels of inflammatory markers [55]. Another experimental study showed that supplementation with prebiotic oligosaccharides (galacto-, fructo- or mannan-) improved memory performance in rodent models [56,57,58]. Interfering with the intestinal microbiota displays an interesting aspect of ameliorating disease symptoms and improving cognitive functions, but it seems to have its limitations as a disease-modifying therapy and in restricting disease progression and neurodegeneration. Further research is needed to fully evaluate the potential of pre- and probiotics for neurodegenerative disease [54,59]. Overall, these findings suggest that intestinal inflammation and dysbiosis may play a role in the pathogenesis of AD, and targeting gut health could represent a disease-modulating therapeutic strategy for AD.

### 2.3. Parkinson’s Disease

Parkinson’s disease is the second most common neurodegenerative disorder characterized by the progressive loss of dopaminergic neurons in the substantia nigra and the presence of intracellular protein inclusions containing α-synuclein aggregates (Lewy bodies), leading to motor and non-motor symptoms, among others. The incidence of PD increases with age and is estimated to affect 2–3% of the population over the age of 65 [60]. While the exact cause of PD is unknown, both genetic and environmental factors contribute to the disease’s pathogenesis. Pathogenic variants in several genes, such as alpha-synuclein (*SNCA*), leucine-rich repeat kinase 2 (*LRRK2*), and Parkinsonism-associated deglycase (*PARK7*), have been associated with rare forms of PD. In addition, aggregation of alpha-synuclein in Lewy bodies and Lewy neurites is a neuropathological hallmark of genetic and sporadic forms of PD [60]. Moreover, environmental factors, such as exposure to environmental toxins (e.g., pesticides) and heavy metals, also increase the risk of developing PD [61]. The diagnosis of PD is based on clinical symptoms and the response to dopaminergic medication, which remains the mainstay of treatment. However, the progression of the disease is inevitable, and there is currently no cure for PD. Research efforts are aimed at identifying disease-modifying therapies and better understanding the underlying molecular mechanisms of PD [60,61]. Of note, recent studies have implicated the involvement of extracellular vesicles (EVs), including exosomes and microvesicles, in the pathogenesis of PD [62]. These vesicles, which are released by various cells in the body, can transport proteins and other molecules between cells and organs and contribute to intercellular and interorgan communication. Within the CNS, EVs are secreted by various cell types, including neurons, astrocytes, microglia, and oligodendrocytes. In the context of PD, EVs derived from neurons and glial cells have been implicated in the propagation of pathological proteins, such as α-synuclein, between cells. Several studies have reported altered levels of EVs in the cerebrospinal fluid (CSF) [63,64,65,66] and blood [65,66,67,68] of PD patients compared to healthy controls. Moreover, these EVs are enriched with α-synuclein [63,68] and other proteins [65] or carrying microRNAs (miRNAs) and other nucleic acids that can modulate gene expression [64,66,67] and are associated with PD pathogenesis. A recent study demonstrated the usage of α-synuclein concentration in plasma EVs to discriminate between PD, healthy controls, and atypical PD, such as dementia with Lewy bodies or progressive supranuclear palsy [69]. Similarly, the latest research has highlighted the detection of pathological α-synuclein conformers from neuron-derived EVs as a potential novel blood-biomarker of PD [70]. Interestingly, α-synuclein aggregates could also be detected within the GI tract of PD patients [71,72]. Moreover, several studies suggest that α-synuclein aggregation and, accordingly, PD pathogenesis can start in the GI tract. Two independent studies described increased α-synuclein aggregates in early and prodromal PD patients in intestinal tissue [73,74]. Moreover, Stockholm et al. highlighted the presence of pathological protein aggregates even several years before the PD diagnosis within the intestine [74]. Additionally, current findings derived from animal models suggest that intestinal inflammation and a pro-inflammatory immune response in the gut can lead to α -synuclein aggregation and promote neuronal damage [75,76]. In a transgenic mice model for PD, immunohistochemical analysis revealed a progressive expression and accumulation of age-dependent α-synuclein in colonic tissue several months prior to the loss of dopaminergic neurons [77]. Multiple studies have suggested that various forms of α-synuclein may be transmitted to the brain via the vagus nerve [78,79,80]. Of particular note, different studies demonstrated the accumulation and the transport of α-synuclein from the intestine to the brain via the vagus nerve. Moreover, this transport via the vagus nerve was associated with motor and cognitive impairment in mice [78,81]. A recent study has indicated that the propagation of α-synuclein pathology from the gut to the brain is more effective in aged compared to young wild-type rats following gastrointestinal injection of aggregated α-synuclein [82]. Moreover, it is worth noting that overexpressed α-synuclein has also been shown to be transmitted from the brain to the ENS, suggesting a bidirectional pathway [83]. Hence, the gut–brain axis represents an important aspect in the development of PD.

### 2.4. Inflammatory Bowel Disease

Intestinal disorders have been associated with neurological alterations. Inflammatory bowel disease (IBD) is an immune-mediated inflammatory disease affecting the GI tract. The two ideotypes of IBD include ulcerative colitis (UC) and Crohn’s disease (CD). CD is characterized by skip lesions, transmural inflammation, granulomas, fistulae, and frequent terminal ileum involvement. Whereas UC—as the second major form of IBD—is featured by continuous colorectal disease, often more prominent distally, with predominant mucosal involvement [84,85]. While IBD primarily affects the gut, it can also cause extraintestinal manifestations, including neurological disorders. For example, IBD patients can experience neuropsychiatric symptoms, such as depression and anxiety. Up to a third of IBD patients are affected by anxiety symptoms and a quarter are affected by depression symptoms, with a higher prevalence of anxiety and depression in patients with an active disease [86,87]. Furthermore, a bi-directional association between MS and IBD has been suggested due to their common epidemiological and immunological patterns [88]. As an example, a comprehensive meta-analysis conducted by Kosmidou et al. revealed a notable 55% elevated risk among patients with MS to develop IBD, alongside a parallel 53% heightened risk of developing MS in individuals already diagnosed with IBD. [89]. A similar analysis performed by Wang et al. evaluating 17 studies described a pooled prevalence of 0.2% of MS in patients with IBD and 0.6% of IBD in patients with MS. Both patient groups (IBD and MS) displayed higher risks of developing additional intestinal or neurological manifestation than healthy controls, but no differences for CD and UC were described [88,89]. Interestingly, current data support the hypothesis that IBD and MS might be genetically linked. Different genes in IBD-associated loci overlap with other immune-mediated disorders, including MS, and shared single nucleotide polymorphisms (SNPs), associated with higher disease risks, were detected for MS and IBD [85,90,91]. Additionally, MS and IBD have similar environmental risk factors, such as vitamin D deficiency, cold climate, socioeconomic status, and smoking [88]. Moreover, alterations in gut microbial compositions have been implicated in the pathogenesis of both diseases. When epithelial cell death becomes dysregulated or excessive, it leads to an increase in intestinal permeability. This heightened permeability, in turn, facilitates the leakage of bacterial antigens into the mucosal and systemic circulation, ultimately predisposing the host to extraintestinal inflammation [17,37,92]. Thus, the gut microbiota, by regulating gut barrier function and immunity, can significantly contribute to the development of both mucosal- and neuroinflammation. In addition to the epidemiological data connecting chronic mucosal inflammation with MS, IBD patients have an increased risk of developing dementia [93,94], AD [95], and PD (especially with increasing age) [96,97]. Moreover, several genetic as well as environmental risk factors are shared between IBD and AD [98,99] or IBD and PD [100,101]. Overall, these findings suggest that IBD patients are more frequently affected by neurodegenerative and neuroimmunological disorders.

## 3. Mechanisms of Microbe-Host Communication

While the importance of the gut microbes was described some time ago for IBD and a variety of other immune and metabolically driven diseases, the essential role of the gut microbiota in CNS inflammation was discovered only a few years ago [102]. To understand why environmental factors derived from the gut microbiota have such a huge impact on host physiology and pathophysiology, it is important to mention that the gastrointestinal tract harbors more than 100 trillion microbial cells belonging to more than 1000 bacterial species [103]. This results in 10 times more microbial than human cells in our body. Accordingly, it is not surprising that the microbiome plays an important role in the pathogenesis of intestinal and extraintestinal diseases.

Humans are constantly exposed to microbes in their environment. Communication between these two players can have different effects depending on factors such as host genetic background, mucosal barrier dysfunction, bacterial virulence, and transmissibility. The host can detect bacteria by signaling through pattern recognition receptors (PRRs) that recognize pathogen-associated molecular patterns (PAMPs) expressed by the microbes, leading to immune cell activation. PRRs are localized either on the surface of the cell, in endosomes, or intracellularly and comprise, e.g., Toll-like receptors (TLRs), C-type lectin receptors (CLRs), and nucleotide-binding oligomerization domain (NOD)-like receptors (NLRs) [104]. In EAE, TLR signaling through myeloid differentiation primary response 88 (MyD88)—an adaptor for TLRs and cytokine signaling—plays an important role in disease progression, as confirmed by the fact that *Myd88*-deficient mice were protected from developing EAE [105,106]. In addition, TLR9 (sensing pathogenic DNA containing CpG motifs and being expressed by monocytes, macrophages, plasmacytoid dendritic cells (DCs), and B cells) and MyD88 are crucial in the modulation of autoimmune processes during the effector phase of EAE [106,107]. TLR9-expressing plasmacytoid DCs, which are an important source of type I interferons (IFNs), produce IFNα in response to TLR9 ligation in early endosomes and are present in the leptomeninges and demyelinating lesions of MS patients [105]. Of note, astrocytes also express TLR9 and activation of this receptor leads to the production of pro-inflammatory mediators that exacerbate tissue damage and neuroinflammation in the CNS [108]. However, human TLR9 appears to play both a protective and deleterious role depending on the MS disease stage [109]. Furthermore, mice lacking the dendritic cell immunoreceptor (DCIR), a CLR involved in the suppression of T cell function, show worsening of EAE, indicating that CLR regulation is important in the development of autoimmune disorders like MS [110]. In addition to this, members of the NLR family that specifically bind peptidoglycans have been identified as both positive and negative regulators of inflammatory responses. Notably, mutations in the NLR family pyrin domain containing 1 (*NLRP1*) gene have been linked with inflammatory disorders, including MS [111,112].

Moreover, TLR activation has also been implicated in the initiation and progression of PD. Several studies have shown increased expression and activation of TLRs, particularly TLR2 and TLR4, in immune cells as well as brain tissue of PD patients and preclinical animal models [113,114,115,116]. The activation of TLRs in microglia, the resident immune cells of the CNS, leads to the production of pro-inflammatory cytokines and chemokines, exacerbating neuroinflammation [117,118]. Interestingly, TLR2 activation in microglia can be triggered by α-synuclein and is an important aspect of PD progression [117,119]. In contrast to this, TLR activation within the intestine influences the intestinal barrier integrity and can promote the aggregation of α-synuclein and its spread within the brain, contributing to the propagation of PD pathology [120,121,122]. Moreover, TLRs can induce the activation of inflammasomes, which are protein complexes involved in the processing and release of pro-inflammatory cytokines, including IL-1β. Inflammasome activation has been implicated in PD pathogenesis, further emphasizing the involvement of TLRs in the disease [123,124]. In the case of AD, TLRs have also emerged as important players in disease progression. For example, alterations of TLR4 signaling are associated with neurotoxic actions [125,126], whereas TLR3 activation can attenuate neuronal loss in an early stage of a preclinical AD model [127]. TLRs, particularly activation of TLRs in microglia and astrocytes, trigger the release of pro-inflammatory molecules, leading to a sustained inflammatory response that can contribute to synaptic dysfunction and neuronal damage [128]. In addition to their role in neuroinflammation, TLRs in AD are involved in the clearance of Aβ plaques [129]. In summary, TLR can have oppositional functions during the pathogenesis of neurodegeneration and neuroinflammation, and it is an important aspect during disease progression.

Inflammation may also occur without a persistent infection through an immune response directed against antigens present in the targeted tissues. These antigenic components could cross-react with self-antigens to change the regulatory state of the host. Various mechanisms based on bacterial antigens have been suggested to clarify how pathogens might trigger autoreactive immune responses. For example, according to the molecular mimicry theory, foreign peptides with sequence similarity to self-peptides can trigger pathogen-derived autoreactive T cells [107]. Of note, in EAE, administration of the encephalitogenic antigen requires co-administration of an adjuvant to enhance its immunogenicity and effectiveness. For example, the complete Freund’s Adjuvant contains heat-killed *Mycobacterium tuberculosis* and activates antigen-presenting cells (APCs) through TLR2 via binding of mycobacterial components that elicit a Th1 response and cause an augmented delayed-type hypersensitivity to self-antigens [130]. Furthermore, superantigens are another mechanism by which bacterial microbes can influence the host immune system. Superantigens, such as staphylococcal enterotoxin B (SEB), can trigger bystander activation and stimulate T cells specific for self-antigens without the need for antigen processing. While the role of SEB in the pathomechanism of MS is currently unknown, it has been associated with the reactivation of EAE. One example is the inoculation of SEB into myelin basic protein (MBP)-immunized mice, resulting in a clinical relapse in those mice that had already recovered from a previous EAE episode [107,131].

In conclusion, pathogens may have an impact on the onset and pathogenesis of neurodegeneration and neuroinflammation either by playing a protective role or by worsening disease manifestation during immunologic maturation.

## 4. Microbial Dysbiosis: Implications for Pathogenesis and Therapeutic Strategies

Colonization of the GI tract starts directly after birth [13,132]. In adults, the GI tract contains an abundant and diverse microbial community with bacterial species belonging to the Firmicutes, Bacteroidetes, Proteobacteria, and Actinobacteria phyla [133,134,135,136]. In contrast to the human genome, which consists of approximately 23,000 genes, the gut microbiome encodes over 3 million genes, producing thousands of metabolites [137]. The diversity of the microbiome is modulated by various host-derived soluble factors, including metabolites (e.g., bile acids), peptides with antimicrobial activity (e.g., defensin and lysozyme), mucin, and sIgA [138,139]. These factors are released not only by epithelial cells but also by mucosal immune cells, and recently, it has been shown that the ENS can modulate the gut microbiota [140]. In turn, gut microbes, their metabolites, and soluble antigens provide immune stimulatory signals regulating innate and adaptive immune responses and, thus, maintain immune homeostasis [13]. This symbiosis is important for the host since the gut microbiota—besides shaping our immune system—also protects the host from invasive pathogens by competing for the same environmental niche, producing neurotransmitters, and processing nutrients such as vitamins and fatty acids [141]. Therefore, they are often referred to as our “hidden metabolic organ” [37].

Numerous clinical studies have highlighted the presence of reduced diversity and disrupted composition of the gut microbiota, known as dysbiosis, not only in mouse models of neuroinflammation and neurodegeneration but also as a common characteristic among patients with PD [142,143,144,145] and MS [22,23,146,147,148,149,150,151]. While dysbiosis has been consistently demonstrated in various clinical and preclinical studies, the existence of a microbiota specifically associated with neuroinflammation or neurodegeneration is still a subject of debate. Additionally, it remains uncertain whether dysbiosis actively modulates inflammatory processes in the CNS or if it is merely a consequence of neuroinflammation/neurodegeneration.

For MS patients, several studies have shed light on the alterations in microbial composition. One of the bacteria that has been discussed intensively is *Akkermansia muciniphila* (*A. muciniphila*), with an enhanced abundance in MS patients [23,147,152,153,154]. Of note, *Akkermansia* is a Gram-negative, strictly anaerobic, oval-shaped, non-motile bacterium that has previously been linked to obesity [155]. Interestingly, *Akkermansia* is known to modulate immune responses and to alter the metabolite pool, specifically butyrate levels within the intestine [156]. Liu et al. demonstrated that *A. muciniphila*, on the one hand, promotes the expansion of Tregs that suppress EAE symptoms [157]. On the other hand, in vitro studies using individual culture extracts of *A. muciniphila* and *Acinetobacter calcoaceticus* showed that these protein lysates were able to promote the polarization of Th1 cells while suppressing Treg differentiation under T cell polarizing conditions [149]. Furthermore, mice colonized with *Akkermansia* isolated from MS patients displayed reduced EAE disease scores linked to IL-17-producing RORγ T cells, suggesting a beneficial role [153]. Moreover, the role of *Akkermansia* in either strengthening or weakening the intestinal barrier by its mucus-degradative function is controversial and discussed [156,158,159,160]. Although there is a strong correlation between mucus-degrading bacteria like *A. muciniphila* and MS, its exact role in MS pathogenesis is still unknown. Currently, it is believed that mucus-degrading microbes have a pro-inflammatory effect only when combined with certain additional gut pathobionts [161,162]. Thus, *Akkermansia* species appear to play a dual role in MS disease, which seems to be context-dependent. Tremlett et al. (by comparing samples of pediatric MS patients to controls) were able to detect that Fusobacteria abundance exhibited a strong positive association with Tregs and that members of the Firmicutes phylum were inversely associated with Th1 cells. They proposed that the depletion of Fusobacteria in pediatric MS cases is associated with a higher risk of relapse [150]. Vice versa, species with a reduced abundance, such as *Bacteroides*, are often reported to have an anti-inflammatory effect on the host’s immune system. For example, Round et al. showed that polysaccharides (PSA)—as a symbiont-associated molecular pattern (SAMP)—of *Bacteroides fragilis* (which was decreased in MS patients [22]) are able to directly activate the TLR2 pathway on Tregs, thereby inducing mucosal tolerance, suppressing antibacterial immune responses via suppressing Th17 cells, and promoting microbial colonization of the gut lumen [163,164]. Oral administration of PSA can prevent mice against EAE by enhancing dendritic cells and, subsequently, Treg activation [165]. The central function of the gut microbiota in the pathogenesis of neuroinflammation has been demonstrated in several preclinical studies. Important findings from these studies could point out that the development of EAE is highly dependent on the presence of microorganisms within the GI tract. In this context, germ free (GF) mice and microbiota-depleted mice exhibit resistance to the onset of EAE [24,166], while monocolonization with, e.g., segmented filamentous bacteria (SFB)—capable of inducing Th17 polarization—can effectively restore disease susceptibility [24]. Preclinical studies have also provided insights into the potential mechanisms underlying the involvement of the gut microbiota in MS. One of the key components is bacterial metabolites. Commensal bacteria-derived metabolites, originating in the gut, can cross the BBB to alter neuroimmunology in the CNS [167]. Cytokines produced in the CNS act in combination with these metabolites to activate anti-inflammatory pathways in CNS-resident glial cells (astrocytes and microglia) to modulate CNS inflammatory mediators and their interaction with neurons and oligodendrocytes [167]. Importantly, such gut–brain communication is highly relevant to disease progression in acute and chronic stages of autoimmune CNS inflammation, as seen in MS patients [168]. These key findings strongly suggest the fundamental role of the gut microbiota in influencing physiological functions and pathological processes in the CNS. In conclusion, it is suggested that the altered microbiota of MS patients promotes pathogenic T cell activation. However, bacteria can be both foes and friends in the context of neuroinflammation [169]. Given the importance of the gut microbiota in MS pathogenesis, interventions that target the gut microbiota may be a promising therapeutic approach for this disease. Strategies such as probiotics, prebiotics, and fecal microbiota transplantation have shown promise in preclinical studies and are currently being investigated in clinical trials [170,171]. For example, Lavasani et al. showed that supplementation of probiotic bacterial species (*Lactobacillus*) can ameliorate disease through immunomodulation on T lymphocytes [172]. Similarly, oral administration of the probiotic strain *Escherichia coli* (*E. coli*) Nissle 1917 reduces susceptibility to neuroinflammation by improving intestinal barrier function in mice [173]. Moreover, administration of *Prevotella histicola* can suppress disease severity in mice with EAE by modulating Tregs [174]. In line with this, MS patients have a decreased abundance of *Prevotella* and *Sutterella*, widely prevalent commensals with mild pro-inflammatory capacity. Of particular note, after treatment with disease-modifying therapies, the abundance of *Prevotella* and *Sutterella* could be enhanced in MS patients [147,175]. In contrast to MS, a higher abundance of *Prevotella* spp. was associated with different inflammatory diseases, such as rheumatoid arthritis [176]. This highlights the complex and variable interplay between bacterial species and various diseases. Understanding the complex interplay between gut microbiota and MS pathology is an important area of research that may ultimately lead to the development of novel treatments for this debilitating disease.

Interestingly, neurodegenerative and neuroinflammatory disorders display similar microbial alterations. A lower abundance of the genera *Roseburia*, *Fusicatenibacter*, *Blautia*, *Anaerostipes* (Lachnospiraceae family), and *Faecalibacterium* (Ruminococcaceae family) was not only detected in samples derived from MS patients but also noted in PD patients or patients with neuromyelitis optica spectrum disorder [162,177,178]. A higher abundance of *Akkermansia* was also detectable in PD patients and not only in patients with MS [23,147,152,178,179]. Moreover, an enriched abundance of the genera *Lactobacillus* and *Bifidobacterium* linked with a decreased abundance of *Faecalibacterium* spp. has been reported for PD patients as well as for patients with IBD [178,179,180]. Surprisingly, *Bifidobacterium* and *Lactobacillus* are commonly considered to be beneficial probiotic bacteria. Indeed, several clinical studies demonstrated an improvement of constipation and motor function in PD patients after administration of probiotics (e.g., *Bifidobacterium* and *Lactobacillus*) [181,182]. Of particular note, the abundance of *Bifidobacterium* and *Lactobacillus* can be modulated via PD medication [143,183,184] and is correlated with clinical inflammatory indicators, such as the percentages of neutrophils and monocytes [185], and associated with active IBD [186]. Moreover, there is evidence that gut dysbiosis can contribute to the accumulation of misfolded α-synuclein protein in the gut, which can then travel to the brain and contribute to the pathology of PD. A recent study demonstrated that immunization with specific α-synuclein epitopes in a mouse expressing a human leukocyte antigen linked to autoimmunity triggers intestinal inflammation and promotes neurodegeneration [187]. At a mechanistic level, it has been proposed that the dysbiotic microbiota may contribute to neurodegeneration in the first step by impacting the integrity of the gut barrier. This disruption in barrier function could lead to increased permeability, creating an environment prone to inflammation and oxidative stress, which in turn may promote the accumulation of α-synuclein in the ENS. Through a prion-like mechanism, this process could potentially propagate across the vagal nerve to the CNS [188]. Recent studies using animal models of neurodegeneration have provided experimental support for this hypothesis [78,189]. Understanding the pathophysiology of the CNS has provided functional evidence of how the gut microbiota influences immune responses, not only within the gut itself but also in the CNS, mediated by microbial-derived metabolites like SCFAs. A preclinical study using α-synuclein-overexpressing mice demonstrated that animals treated with SCFAs through gavage exhibited significantly impaired performance in several motor tasks, and α-synuclein aggregation was more pronounced in their brains compared to untreated mice. These effects may be attributed to the promotion of microglial morphology to a more active state within affected brain regions [190]. However, fecal microbiota transplantation from PD patients has been found to exacerbate disease progression, indicating the presence of specific disease-promoting microbes [190]. Supporting this notion, Li et al. confirmed that PD patients exhibit alterations in the microbiota that correlate with disease progression, including a continuous decrease in fiber-degrading bacterial strains and an increase in pathobionts. These changes likely result in reduced SCFA production and increased production of endotoxins and neurotoxins [191]. Notably, growing evidence indicates that fecal microbiota transplantation from healthy donors, as well as administration of butyrate in animal models of PD, improves motor impairment and alleviates dopamine deficiency [192,193,194,195,196]. Another recent investigation demonstrated the significant impact of the intestinal microbiota in a genetic mouse model that mimics PD pathology. While pathogenic variants in PTEN-induced kinase 1 (*PINK1*) and parkin (*PARK2*) genes in PD patients are associated with disease progression, *Pink1*-knockout mice do not develop PD-like symptoms [197]. However, when these mice were infected with a Gram-negative bacterium (*Citrobacter rodentium*) causing mild colitis during early life, PD-like symptoms were triggered later in life [198]. Overall, gut microbiota is an emerging area of research in the field of PD and has the potential to provide new insights into disease pathogenesis and novel therapeutic strategies.

In contrast to PD, a decrease in certain beneficial bacterial species, such as *Bifidobacterium* and *Lactobacillus*, was described for AD patients. Moreover, AD is associated with a higher abundance of Bacteroidetes and pro-inflammatory *Escherichia* and *Shigella* [48,49,199,200]. Exploring the mechanisms through which the intestinal microbiota influences AD progression could uncover a valuable therapeutic target capable of controlling multiple disease mechanisms. Initial studies highlighted the profound impact of microbial alterations on AD-related pathology [201,202,203]. In preclinical mouse models of amyloidosis, administration of antibiotics (abx) [201,202,204,205,206] or GF conditions [36,203,207] leads to reduced amyloidosis and microglial activation. Additionally, abx treatment also enhanced the presence of anti-inflammatory regulatory T cells in the brain and blood [202]. Moreover, microbial alterations are associated with modulated permeability of the BBB and neuroinflammation linked to AD pathogenesis. Enhanced permeability of the gut and BBB allows large amounts of amyloids and LPS to circulate within the cardiovascular system and CNS and to modulate various signaling pathways, including the production of pro-inflammatory cytokines associated with AD [51,208]. Furthermore, gut dysbiosis-induced peripheral immune responses can propagate bacterial and pro-inflammatory signals to the brain [198,209]. Moreover, a recent pilot study conducted with a small sample size and randomized, double-blind design in AD patients suggested that a modified Mediterranean ketogenic diet could potentially alleviate AD symptoms by modulating SCFAs. Specifically, the associated reduced levels of fecal lactate and acetate and increased concentration of propionate and butyrate might induce or be linked with the improvements in AD biomarkers in the cerebral spinal fluid [210]. Zhang et al. conducted an animal study using APP/PS1 (amyloid precursor protein/presenilin 1) transgenic AD mice, which revealed lower concentrations of butyric acid in both feces and the brain, along with a decrease in the abundance of *Butyricicoccus pullicaecorum*, a butyrate-producing bacterium. These changes may contribute to cognitive decline in AD [211].

In summary, gut dysbiosis is an emerging area of research in the field of neuroinflammation and degeneration, and understanding the complex interplay between gut microbiota and disease pathology may lead to the development of new therapeutic strategies for this devastating disease.

## 5. Bacterial Cell Wall Components and Bacterial Extracellular Vesical as Novel Aspect within the Microbe–Host Interaction

Moreover, it was shown that dysbiosis enhances the permeability of the intestinal epithelial barrier and exposes the host not only to higher levels of microbial metabolites but also to an increased amount of cell wall components, such as LPS, different peptidoglycan (PG), lipoteichoic acids (LTA), and bacterial extracellular vesicles. Bacterial cell wall components and bacterial vesicles may have a significant impact on the pathogenesis of neurological diseases.

### 5.1. LPS as Inducer of Neuroinflammation and Neurodegeneration

The cell wall component which is most abundant in Gram-negative bacteria is LPS. In dysbiotic microbiota, LPS is more abundant, contributing to the dysfunction of the mucosal barrier. LPS can also affect other tissues, including the CNS if its plasma levels experience an increase [212]. Interestingly, LPS can either directly or indirectly interact with the BBB [213]. Early work by Wispelwey et al. showed that LPS—when entering the systemic circulation—can disrupt the BBB [214]. In this context, LPS has direct effects on tight junction regulation, thereby increasing the permeability of the BBB when applied to monolayers of brain endothelial cells [215]. In addition, LPS can interact with CNS-resident cells by regulating the production of inflammatory proteins, such as the induction of reactive oxygen species (ROS), which can mediate microglial activation, among other effects. ROS have further been reported to be relevant in MS disease pathogenesis, serving as mediators associated with demyelination and axonal damage. Interestingly, MS patients show elevated levels of oxidative stress markers (such as ROS), together with a systemic antioxidant deficiency. Accordingly, the resulting oxidative stress has been associated with the course of disease, implying that an impaired balance between ROS and antioxidants may be linked to relapse in patients with MS [212].

In addition to MS, several studies have linked AD neuropathology to the presence of LPS in the brain. Research has demonstrated the coexistence of LPS and Aβ1–40/42 within amyloid plaques located in both gray and white matter of AD-affected brains [216]. Another study revealed the abundance of LPS in the neocortex and hippocampus of AD brains, along with a notable binding of LPS to the periphery of cell nuclei within the AD brain [217]. Furthermore, LPS has been detected in lysates obtained from the hippocampus and superior temporal lobe neocortex of AD-affected brains [218]. In addition to these clinical studies, research conducted on animal models has demonstrated that the inflammation triggered by LPS can mimic certain features of PD. Mechanistic studies have delineated that LPS promotes the activation of microglia, resulting in the release of various neurotoxic substances. Additionally, the presence of damaged neurons can trigger reactive microgliosis, which contributes to the progressive degeneration of dopaminergic neurons associated with PD. These include significant activation of microglia and the specific degeneration of dopaminergic neurons in the nigrostriatal system [219,220,221,222,223]. Within the CNS, it has been observed that systemic administration of LPS leads to an increase in the expression of the CD14 receptor on certain cell populations, particularly microglia in the brain [224]. Subsequently, microglia were identified as the primary cells in the brain that respond to LPS. LPS interacts with TLR4 on microglia, triggering their activation and ultimately causing harm to neurons [225,226]. Moreover, several TLRs (TLR1, TLR2, and TLR4) are expressed by human neurons to detect not only LPS but also further bacterial cell wall components, such as LTA [227,228]. Neuronal death, which occurs through caspase-3-dependent apoptosis, is induced by pro-inflammatory cytokines released in response to PG, LTA, and LPS [229]. Neurons in specific brain regions, such as the hippocampus, prefrontal cortex, and cerebellum, exhibit high levels of peptidoglycan recognition protein 2 (PGLYRP2) and NOD1, enabling them to recognize and differentiate muropeptides derived from both Gram-positive and Gram-negative bacteria [230]. PGLYRP2 binds to the bacterial cell wall to cleave the stem peptide, while NOD1 activation leads to the production of pro-inflammatory cytokines (IL-1β, TNF, IL-6) [231].

### 5.2. Bacterial Extracellular Vesicles as Promoter of Neurodegenerative Diseases

Interestingly, increasing evidence indicates an alternative horizontal communication system between bacteria and their host. This system operates alongside direct host-microbe interaction, involving the release of bacterial-derived functional molecules via bacterial extracellular vesicles (BEVs), thereby facilitating an indirect form of host-microbe interaction [232]. Like most cells of all domains of life, bacteria release 40–400 nm-sized membrane vesicles into their extracellular environment during their normal life cycle. These BEVs are spherical, bilayered proteolipids enriched with proteins, lipids, nucleic acids, metabolites, and virulence factors and are released by both pathogenic and commensal bacteria. The content of these vesicles can vary depending on the bacterial species and growth conditions. They are similar to EVs released by eukaryotic cells but differ in biogenesis and content. Major forms of host-derived EVs include exosomes and microvesicles, which have been suggested to play crucial roles in various physiological and pathological processes. BEVs play an essential role in bacterial communication, adaptation, and virulence, but recent findings in this field also suggest a pathophysiological role of BEVs in both bacteria–bacteria as well as bacteria–host interactions [233,234,235,236]. BEVs transport cargo such as the biological material found within the parental bacterium, including nucleic acid, PG, virulence factors, communication compounds, immune-modulatory compounds, etc., but without replicative mimics. Depending on the specific biogenesis pathway and origin, several forms of BEVs can be separated, and each group has specific characteristics and functions. For example, one type of BEVs produced by Gram-negative bacteria by pinching off the outer membrane are termed outer membrane vesicles (OMVs) and contain, in contrast to other BEVs, no nucleic acid (the different types of BEVs are well reviewed in [235]). These cargos play a crucial role in microbial pathogenesis since they are involved in the invasion of the host cell membrane, membrane fusion, the production of biofilms, and the transfer of virulence factors, as well as receptors and antibiotic-resistance proteins [233,235]. Moreover, BEVs were shown to activate both innate immune cells like macrophages, DCs, and microglia, as well as adaptive immune cells like T and B cells in distant organs, once BEVs are delivered to the systemic circulation [237].

Of particular note, human cell-derived EVs have been reported to be increased in patients with MS compared to healthy controls. Furthermore, they have been described to play an important role in MS development, particularly in BBB weakening, activation of immune cells during relapses, migration through the BBB, and spreading of inflammation in CNS tissue [238]. Since human cell-derived EVs play an active role in the pathophysiological development of MS, it is of great interest to determine the impact of BEVs on MS. Although gut-derived BEVs were shown to enter the systemic circulation, it is currently not known if they can also pass through the BBB and enter the brain. The restricted permeability of the BBB limits the passage of molecules and cells from the bloodstream into the CNS under normal conditions, thus protecting the CNS from potentially harmful substances and making it an immune-privileged organ. However, a recent study by Bittel et al. revealed the transfer of gut bacteria-derived BEVs to a wide range of close and distant host organs, including the brain, in mice [232]. In support of these findings, several other groups followed and further confirmed that BEVs are able to cross the BBB and deliver various molecules to the CNS [232,239,240]. LPS is a major cause of neuroinflammation and neurodegeneration, but only minor amounts of free LPS are able to cross the BBB [241]. Crossing the BBB by BEVs may represent an explanation for the detection of elevated levels of LPS in the CNS of patients suffering from neurodegenerative disorders [216]. The impact of BEVs on CNS function can occur through various mechanisms [242]. While the presence of LPS on BEVs likely contributes to the observed neuroinflammation after BEV administration, the extent of neuroinflammation caused by BEVs exceeds the inflammation, which can be attributed solely to equivalent amounts of LPS [239]. Similar results were also observed for leukocyte adhesion on the endothelium but also in the context of the systemic inflammatory response syndrome [243,244]. This implies additional mechanisms mediated by RNA or protein cargo besides LPS-dependent functions. For example, BEVs derived from *Porphyromonas gingivalis* are densely packed with virulence factors and gingipains (specific bacteria proteinases) [245,246]. These BEVs are connected with tissue destruction in periodontal diseases but are also assumed to promote BBB damage and AD [242,246,247]. Gingipains displayed neurotoxic effects in vitro as well as in murine in vivo models and were identified in the brains of patients with neurodegenerative diseases associated with ubiquitin and tau pathology [248,249]. Furthermore, BBB permeability can be enhanced by BEVs, potentially facilitating other BEVs or bacterial products to access the CNS and influence its function [242,250]. For instance, enterohemorrhagic *E. coli* (EHEC) generates the hemolysin toxin in both free and BEV-associated forms, with the latter showing superior activity and stability. Notably, EHEC BEVs seem to operate as targeted transporters for delivering hemolysin to brain endothelial cells. This transport occurs independently of the presence of hemolysin toxin, unlike the binding of BEVs to erythrocytes, which requires hemolysin. Consequently, BEV-associated hemolysin is likely responsible for detrimental effects on brain microvascular endothelial cells during EHEC infection, in contrast to free hemolysin. This phenomenon could contribute to the neurological manifestations observed in cases of hemolytic uremic syndrome caused by EHEC [242,251,252,253]. These data strongly indicate several mechanisms of BEVs by which they might contribute to neurodegeneration and neuroinflammation, including RNA or protein cargo as well as virulence factors.

Once inside the brain, BEVs have the capacity to directly modify neurological function and trigger pathological changes. Recent studies have demonstrated that BEVs possess the capacity to induce neuroinflammation and influence neuronal function—highlighting their potential neurotoxicity in the context of neurodegenerative diseases [240,247,249,254,255,256,257]. Activation of microglia and astrocytes by virulence factors like LPS, PG, and proteins provided by BEVs elicit the release of inflammatory cytokines and chemokines [240,254,255,256]. Interestingly, the metabolic composition of serum BEVs exhibits significant disparities between AD patients and healthy individuals [258]. In a murine model of AD, the blood BEV profile was found to correlate with the gut microbiome profile, suggesting that gut microbiome-derived BEVs from various bacterial species can effectively enter the bloodstream [259]. To demonstrate the direct involvement of BEVs in AD pathogenesis, Wei et al. isolated BEVs sourced from the gut microbiome of healthy individuals and AD patients and treated mice via tail vein injection with these BEVs. Both BEV groups were able to enter the brain, but only BEVs derived from AD patients weakened the BBB significantly and were able to activate microglia. Mechanistically, they could show that BEVs induce hippocampal neuroinflammation, increase tau hyperphosphorylation via GSK-3B, and induce cognitive deficits in mice [240]—all characteristic features of AD. Notably, advanced age stands as one of the primary risk factors for AD [41]. Aging is associated with an increase in the BBB as well as intestinal permeability, which, in turn, has been linked to elevated blood levels of host and bacterial vesicles [260,261,262,263]. When orally administered to mice, BEVs from *Paenalcaligenes hominis* (*P. hominis*)—bacteria that are found at significantly higher levels in the gut microbiota of elderly individuals—triggered microglia activation, neuroinflammation, and cognitive impairment [239]. Intriguingly, cognitive impairment, neuroinflammation, and hippocampal BEV accumulation were significantly inhibited by vagotomy, suggesting that certain *P. hominis* BEVs transit to the brain and potentially signal through the vagal nerve [239]. This observation aligns with previous studies, strongly highlighting the vagus nerve as an important player within the gut–brain axis [264]. These findings collectively establish a connection between age-related alterations in the microbiome and the increased risk of AD with advancing age while also offering further support for the concept that BEVs can infiltrate the brain and contribute to neurodegenerative disease pathology. Although the primary natural role of Aβ remains uncertain, emerging evidence indicates its potential role as an antimicrobial peptide (AMP) released in response to brain infections [265,266]. It is assumed that not only bacteria but also BEVs are able to promote the production of AMPs [267]. Indeed, studies using a murine AD model highlighted that the gut microbiome can drive Aβ production [203]. Moreover, *Helicobacter pylori* (*H. pylori*) filtrate, potentially containing BEVs, prompted Aβ production and cognitive impairments in a murine AD model [268]. Xie et al. demonstrated that BEVs derived from *H. pylori* are able to cross the intestinal as well as the BBB barrier, activating astrocytes, which subsequently leads to neuronal dysfunction and increased Aβ pathology [254]. Further investigation into the link between BEVs and AD pathology, as well as further neurological disorders, will be essential for deciphering the underlying pathomechanisms and revealing future therapeutic strategies.

In addition to their potential neurotoxicity, research has unveiled the protective role of BEVs in the context of neurodegenerative diseases. Accordingly, multiple studies have highlighted the capacity of BEVs to represent not only therapeutic targets but also enable therapy strategies and therapeutical benefits [242,269,270]. In the context of the gut–brain axis, previous studies have demonstrated that BEVs originating from commensal or probiotic gut bacteria can suppress intestinal inflammation, modulate immune responses, enhance cognitive function, and mitigate neuroinflammation in preclinical mouse models [271,272,273,274,275,276]. These findings suggest the potential therapeutic utility of BEVs. While the cautious use of BEVs as therapeutics is essential due to their toxic potential, their protective and immunomodulatory attributes offer novel prospects for disease treatment in the context of intestinal inflammation as well as neurodegeneration. Notably, specific BEVs derived from distinct bacterial strains have been shown to enhance neuronal viability and function in neurodegenerative disease contexts. Specifically, BEVs originating from the probiotic *Lactobacillus plantarum (L. plantarum*) were associated with beneficial neurological effects. *L. plantarum* BEVs were found to enhance the expression of brain-derived neurotrophic factor (BDNF). These BEVs also displayed the ability to counteract and reverse the decrease in hippocampal *Bdnf1/4* and neurotrophin 4/5 (*Nt4/5*) expression caused by restraint stress in HT22 hippocampal neurons and in the mouse brain, subsequently alleviating neuronal function and depressive-like behaviors. The antidepressant-like effects observed with *L. plantarum* BEVs resembled those induced by the antidepressant imipramine and were stably maintained [276]. Moreover, these BEVs were able to reduce neuronal death in vitro, as well as in vivo, in the context of ischemic stroke by targeting apoptosis by specific miRNA [271]. To achieve a comprehensive understanding of the underlying mechanisms that define the defensive attributes of BEVs and to explore their therapeutic prospects for neurodegenerative diseases, further investigation is warranted.

In summary, the gut–brain axis stands as a pivotal avenue for unraveling neuroinflammation and neurodegenerative pathologies. Intestinal microbes influence both the enteric and central nervous system. Beyond generating microbial metabolites and neurotransmitters, the release of BEVs represents a novel and crucial dimension within host-microbe communication. Further research is required to fully understand the contribution of microbial metabolites and BEVs in the context of neuroinflammation and neurodegeneration.
